# Montelukast in Adenoid Hypertrophy: Its Effect on Size and Symptoms

**Published:** 2015-11

**Authors:** Farshid Shokouhi, Ahmad Meymaneh Jahromi, Mohamad Reza Majidi, Maryam Salehi

**Affiliations:** 1 *Department of Otorhinolaryngology,Faculty of Medicine, Mashhad University of Medical Sciences, Mashhad, Iran.*; 2 *Sinus and Surgical Endoscopic Research Center, GhaemHospital, Faculty of Medicine, Mashhad University of Medical Sciences, Mashhad, Iran.*; 3 *Department of Community Medicine, Faculty of Medicine, Mashhad University of Medical Sciences, Mashhad, Iran. *

**Keywords:** Adenoid hypertrophy (AH), Montelukast, Mouth breathing, Sleep discomfort, Snoring

## Abstract

**Introduction:**

Adenotonsillar hypertrophy (AH) is considered the most common cause of upper respiratory tract obstruction among children. It results in a spectrum of symptoms from mouth breathing, nasal obstruction, hyponasal speech, snoring, and obstructive sleep apnea (OSA) to growth failure and cardiovascular morbidity. Adenotonsillectomy is a typical strategy for patients with AH, but may lead to serious complications such as bleeding (4–5%) and postoperative respiratory compromise (27%), especially among young children, as well as recurrence of adenoid tissue (10–20%). Thus, non-surgical therapies have attracted considerable attention as an alternative strategy. The inflammatory mechanism proposed for AH has lead to the use of anti-inflammatory drugs to manage this condition. The present study aimed to evaluate the effect of chewable tablets of montelukast, a cysteinyl- leukotriene receptor antagonist, in children with AH.

**Materials and Methods:**

Sixty children between the ages of 4–12 years with >75% choanal obstruction on primary nasal endoscopy were recruited in this randomized, placebo-controlled trial and randomly divided into two groups. The study group was treated with montelukast 5 mg daily for 12 weeks while the control group received matching placebo for the same period of time. A questionnaire was completed by each child’s parent/guardian to assess the severity of sleep discomfort, snoring, and mouth breathing before and after the intervention.

**Results:**

Adenoid size decreased in 76% of the study group compared with 3% of the placebo group after 12 weeks. A statically significant improvement was observed in the study group compared with the placebo group in terms of sleep discomfort, snoring, and mouth breathing. The symptoms average total score dropped from 7.7 to 3.3 in the study group, while in the placebo group the total score changed from 7.4 to 6.7.

**Conclusion:**

Montelukast chewable tablets achieved a significant reduction in adenoid size and improved the related clinical symptoms of AH and can therefore be considered an effective alternative to surgical treatment in children with adenoid hypertrophy.

## Introduction

Adenoid hypertrophy (AH) is a common disorder among children, with a prevalence of 2–3%. The adenoid is one of the most important parts of the Waldeyer tonsillar ring located in the nasopharyngeal area. Because of its special location, especially in relation to the posterior choanae and Eustachian tube, it is often the source of many health problems of childhood.

The adenoid is very small at infancy but starts growing in the first 4 years of life due to immune system development. Untreated AH may lead to snoring, sleep discomfort, mouth breathing, ear problems, failure to thrive, pulmonary hypertension, and craniofacial anomalies (1). However, in a patient with AH symptoms, diagnosis can be challenging. There are different recommendations for the diagnosis of AH, such as lateral neck X-ray, videofluo- roscopy, palpation, and nasal endoscopy; yet the role of each diagnostic technique is still a controversial issue. In most recent studies, lateral neck X-ray and nasal endoscopy have been suggested as the best modalities for the diagnosis of AH (2).

Adenoidectomy is a common surgical procedure in childhood but, despite the precise selection of patients for surgery possible today, it may result in postoperative complications such as immediate and late bleeding (4–5%) and recurrence of adenoid tissue in 10–20% of cases (1). The risk of anesthesia and other surgically-related morbidities, high costs and the absence of the child from school have further highlighted the need for medical treatment in AH.

Importantly, serum titer of immuno- globulins M, G, and A (IgM, IgG and IgA) in patients having undergone adenoton- sillectomy have been observed to be lower than in healthy controls (3). Similar to adults, children with AH develop systemic inflammation represented by an increase in C-reactive protein. Interestingly, this is correlated with cognitive and cardiovascular morbidity, which decrease following adeno- tonsillectomy.

Leukotrienes are key inflammatory mediators in the respiratory system. These lipid mediators are involved in the pathogenesis of childhood diseases such as asthma. They are also systemically and locally involved in the process of inflammation in children with AH.

Human cysteinyl-leukotriene receptor-1 expression is elevated in the tonsillar tissues of children with obstructive sleep apnea (OSA). Accordingly, cysteinyl-leukotriene receptor-1, which interacts with leukotrienes and mediates the inflammatory pathway, was over expressed in adenotonsillar cells and tissues derived from children with AH (4). Thus, anti-inflammatory agents with a safe therapeutic profile may provide an interventional alternative to adenotonsillectomy.

Montelukast is an oral, bioavailable, cysteinyl-leukotriene receptor antagonist that is effective, safe, well tolerated and approved by the US Food and Drug administration (FDA) for preventive therapy of the inflammatory component in asthma and allergic rhinitis in children aged ≥1year. Furthermore, montelukast has not induced tolerance in long-term studies (5,6).

The purpose of this study was to assess the hypothesis that montelukast therapy might lead to improved nocturnal symptoms, lifestyle, anatomic characteristics, as well as endoscopic and radiologic results in children with AH.

## Materials and Methods

This double-blind, randomized, placebo-controlled clinical trial was performed from April 2013 to September 2014 in the Otorhinolaryngology Clinic of Imam Reza Educational Hospital, Mashhad, Iran. In total, 60 children were recruited among all pediatric patients referred for snoring evaluation. At least 30 patients/group were required to establish an improvement of at least 25% in the outcome parameters measured (such as grading in endoscopy and adenoid nasopharyngeal ratio in radiography). Diagnostic tests included a clinical evaluation, lateral neck radiography, and nasal endoscopy. The inclusion criteria were as follows: children >4 and <12 years of age with habitual snoring and grade 3 or greater nasopharynx obstruction on endoscopy examination and 50% or more in A/n ratio in radiographic studies.

Children with the following criteria were excluded from the study: obesity defined as BMI >1.645 (95%), craniofacial, neuromuscular, syndromic, or defined genetic abnormalities, current or previous use of montelukast, acute upper respiratory tract infection, use of any corticosteroids or antibiotics within 4 weeks preceding the initial sleep study, and any child having undergone adenotonsillectomy in the past.

Children were recruited by a single otorhinolaryngologist and were randomly assigned to the study or control groups (n=30). The study group received montelukast (Aboreyhan Company, Iran), 5 and 10 mg per day for children <6 and >6 years of age, respectively, whereas placebo tablets with the same shape, color and dosage were prescribed for the control group. The study investigators were blinded to group assignments. All parents were instructed to give the tablets at bedtime. Upon completion of the 12-week therapeutic course, patients underwent a second nasal endoscopy exam besides lateral neck radiographic study. During the study period the children’s parents were contacted monthly by the investigators to evaluate their compliance and the drug’s potential side effects.

The study protocol was approved by the Research Council of Mashhad University of Medical Sciences and an informed consent form was signed by the child’s parent/guardian prior to study entrance.

***Lateral Neck Radiographic Study***

For assessment of airway patency, lateral neck radiographs were performed using the standard technique in the Radiology Department of Imam Reza Hospital. The neck was extended, and the patient was instructed to breathe through the nose with the mouth closed (Fig. 2). 

Adenoidal/nasopharyngeal ratio was measured according to the method described by Fujioka and colleagues (7). Fujioka described the A/N ratio for measurement of the obstruction in 1979 (8).

Lateral neck radiography was performed once at study initiation and once again after the 12-week therapeutic course.

**Fig 1:**
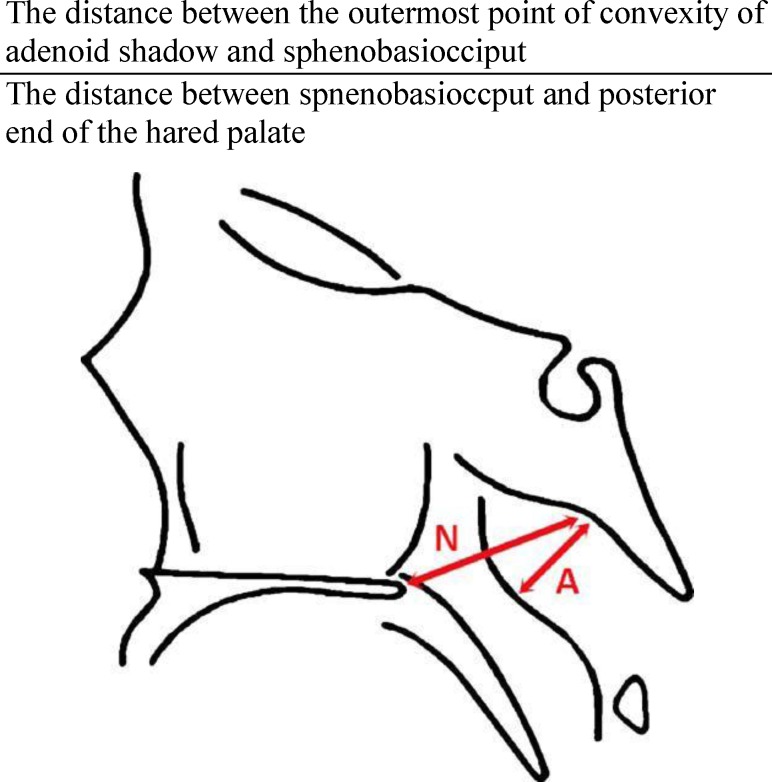
Calculation of the A/N ratio

**Fig 2:**
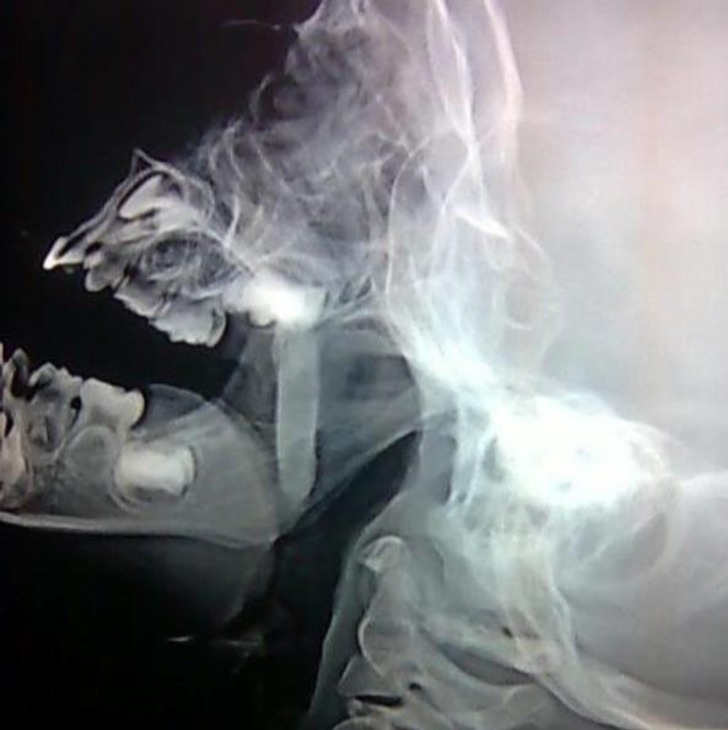
Lateral neck radiography *Nasal Endoscopy*

Nasal endoscopy (using a 2.7-mm Karl Storz [Germany] 0 rigid endoscope) was used to obtain a full choanal image by the same otorhinolaryngologist in all evaluations. Before performing nasal endoscopy, topical anesthesia and vasoconstriction were administered in all patients using a topical solution consisting of 5% xylocaine and 0.5% phenyl ephedrine without any sedation.

The amount of obstruction was categorized using the method of Parikh, which is based on the anatomical relationship between surrounding anatomical structures such as torus tubarius, vomer, and the soft palate (grade 0 = none, grade 1 = torus tubarius, grade 2 = torus tubarius and vomer, and grade 3 = vomer and soft palate). Patients underwent nasal endoscopy both before and after the study (9,10).

*Data Analysis*

Results are presented as mean ± SD, unless stated otherwise. The primary outcome measures were scores for snoring, open-mouth breathing, and sleep discomfort. Secondary outcome measures were the adenoid size estimate based on endoscopy and lateral neck radiography (11). 

All numeric data were subjected to statistical analyses with either t-tests or Mann-Whitney test. Other statistical tests were used wherever appropriate. P<0.05 was considered statistically significant.

## Results

In total, 60 children fulfilling the inclusion criteria were studied. There were no withdrawals, and no side effects occurred in any of the patients (Table. 1).

**Table 1:** Demographic characteristics of the treatment and placebo groups at study entrance

		**Montelukast (n=30)**	**Placebo (n=30)**
Sex	Male	19	15
	Female	11	15
Age (yrs)		6.9±2.33	6.83±2.36
Weight (kg)		22.30±6.798	22.26±6.13

The study variables were measured both by clinical and paraclinical methods. The main studied symptoms were snoring, mouth breathing and sleep discomfort. Based on symptom severity, a score of 0–2 was given as follows: symptoms never existed: 0; occasional symptoms: 1; present at most times: 2; always present: 3. 

**Fig3:**
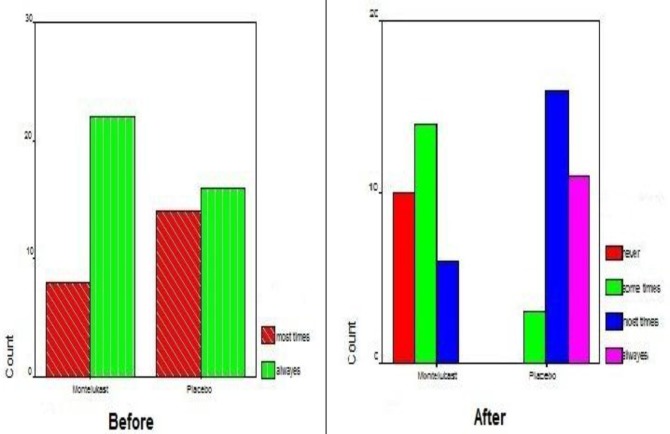
Snoring scores in the study and control groups before and after treatment

According to the Mann-Whitney test, no difference was observed in snoring between the two groups (P=0.111). The mean score in each group was 3. However, following treatment a significant difference was revealed between the two groups (P<0.007) (Fig. 3).

**Fig4:**
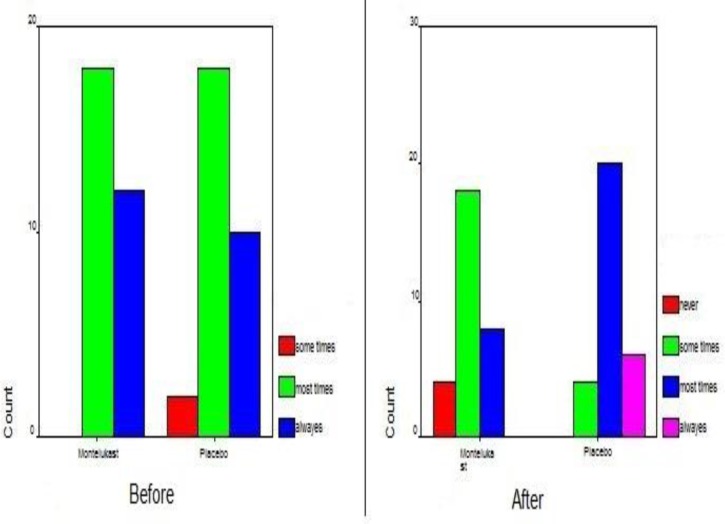
Sleep discomfort scores in the study and control groups before and after treatment

Regarding sleep discomfort, no meaningful difference between the two groups was observed at study initiation (P=0.408). However, following treatment the difference was statistically significant (P<0.0001) (Fig.4).

**Fig 5:**
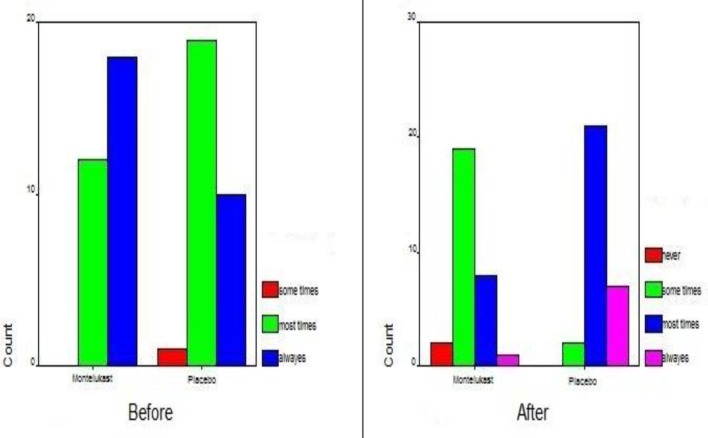
Mouth breathing score in the study and control groups before and after treatment

The obtained results were similar for mouth breathing; showing a strongly meaningful difference only after the therapeutic period (P=0.33 vs. P<0.0001) (Fig. 5). For this symptom, the mean score of the treatment group was higher at study initiation, yet the small difference was not statistically important (Fig. 5).

**Fig 6:**
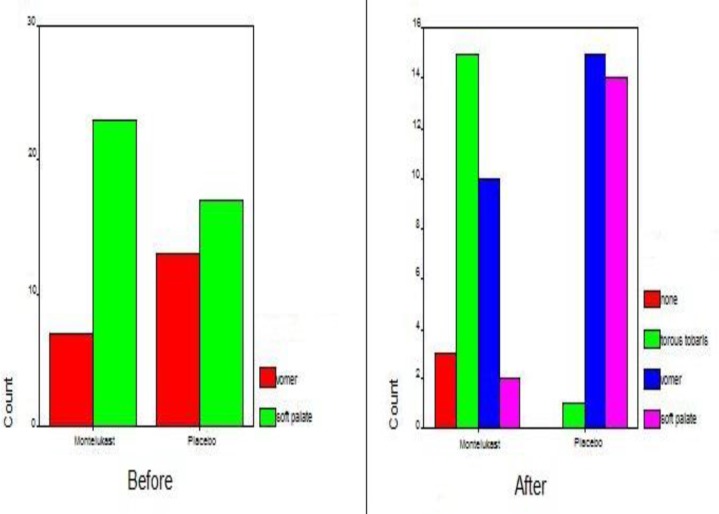
Endoscopic results in the study and control groups before and after treatment

The patients’ symptoms were also studied by the means of nasal endoscopy and lateral neck radiography before and after the treatment course. The mean score between the two groups showed a meaningful difference before the treatment (P=0.03); yet a more significant difference was observed after the treatment course (P<0.0001)(Fig. 6).

In radiographic studies the response to treatment was described as a reduction of ≥25% in adenoid size. The response rate was 76% in the treatment group compared with 3.3% in the placebo group.  A P<0.0001 was considered statically significant.

Cohen's kappa coefficient, a measure of agreement, was used for comparison of these two paraclinic tests in relation to the clinical symptoms. This test revealed a value of 0.55 for radiography and 0.8 for endoscopy. Therefore, endoscopy had a stronger correlation with the patient’s symptoms and his/her general condition (Table. 2).

**Table 2:** Para clinic findings before and after treatment in the two studied groups.

	**Montelukast**		**Placebo**	
	Before	After	Before	After
Endoscopy (score)	3.7667±0.43018	2.3667±0.76489	3.5667±50401	3.4333±0.56832
Radiography (%)	±87.23±8.97	±51.33±5.91	81.16±7.27	77.83±9.06

## Discussion

AH is a common disease of childhood and is the cause of most surgical procedures during the early years of life.

This study showed that montelukast oral chewable tablets, administered over a period of 12 weeks in children with AH, effectively alleviated the severity of snoring, sleep discomfort, and mouth breathing as well as reducing the size of the adenoid tissue. Furthermore, this treatment was not associated with any side effects and was well tolerated by all participants.

Significant reduction in adenoid size was confirmed through nasal endoscopy and lateral neck radiography. Despite certain difficulties in performing endoscopy, it has a stronger correlation with clinical symptoms (10). In a study by Goldbart et al. in 2012 (13), montelukast was used for the treatment of OSA symptoms in 40 children between 4–12 years of age. Twenty children were given chewable tablets of montelukast (4 mg for children <6 years of age and 5 mg for older children) while the other 20 received placebo; both for 12 weeks.

 A significant improvement (>50%) was observed in polysomnographic parameters and the A/N ratio in radiography was reduced from 81% to 57%. The authors recommended this treatment for mild degrees of OSA. The difference in the obtained results might be due to the different drug dosages used (5 mg for children under 6 years and 10 mg for older children in our study) or the difference in the severity of symptoms at study entrance. Our patients had moderate–to-severe symptoms, whereas in the mentioned study the symptoms were mild to moderate. 

Overall, montelukast can be recommended as an alternative to surgery with the aim of preventing postoperative complications.

## Conclusion

There is increasing evidence that AH may be associated with significant cognitive, behavioral, and vascular morbidity. It may even have a major impact on quality of life and healthcare costs (12). The results of this study support the introduction of a leukotriene modifier as a novel and safe therapeutic alternative for the treatment of children with symptoms of AH. However, before this approach can be accepted as a medical standard of care, large-scale studies are warranted to further reinforce our findings.

## References

[1] Shirley WP, Wooley AL, Cummings et al Pharyngitis and Adenotonsillar Disease. Otolaryngology: Head and Neck Surgery.

[2] Saedi B, Sadeghi M, Mojtahed M, Mahboubi H (2011). Diagnostic efficacy of different methods in the assessment of adenoid hypertrophy. Am J Otolaryngol.

[3] Ikincioğullari A, Doğu F, ikincioğullari A, Eğin Y, Babacan E (2002). Is immune system influenced by adenotonsillectomy in children. Int J Pediatr Otorhinolaryngol.

[4] Dayyat E, Serpero LD, Kheirandish-Gozal L, Goldman JL, Snow A, Bhattacharjee R (2009). Leukotriene pathways and in vitro adenotonotonsillar cell proliferation in children with obstructive sleep apnea. Chest.

[5] Berlucchi M, Salsi D, Valetti L, Parrinello G, Nicolai P (2007). The role of montelukast in the treatment of adenoidal hypertrophy in the pediatric age group: preliminary results of a prospective, randomized study. Pediatrics.

[6] Mizutani N, Nabe T, Imai A, Sakurai H, Takenaka H, Kohno S (2001). Markedly increased nasal blockage by intranasal leukotriene D4 in an experimental allergic rhinitis model: contribution of dilated mucosal blood vessels. Jpn J Pharmacol.

[7] Cohen D, Konak S (1985). The evaluation of radiographs of the nasopharynx. Clin Otolaryngol Allied Sci.

[8] Fujioka M, Young LW, Girdany BR (1979). Radiographic evaluation of adenoidal size in children: adenoidal-nasopharyngeal ratio. AJR Am J Roentgenol.

[9] Parikh SR, Coronel M, Lee JJ, Brown SM (2006). Validation of a new grading system for endoscopic examination of adenoid hypertrophy. Otolaryngol Head Neck Surg.

[10] Caylakli F, Hizal E, Yilmaz I, Yilmazer C (2009). Correlation between adenoid-nasopharynx ratio and endoscopic examination of adenoidhypertrophy: a blind, prospective clinical study. Int J Pediatr Otorhinolaryngol.

[11] Goldbart AD, Goldman JL, Veling MC, Gozal D (2005). Leukotriene modifier therapy for mild sleep-isordered breathing in children. Am J Respir Crit Care Med.

[12] Kuhle S, Urschitz MS (2011). Anti-inflammatory medications for obstructive sleep apnea in children. Cochrane Database Syst Rev.

[13] Goldbart AD, Greenberg-Dotan S, Tal A (2012). Montelukast for children with obstructive sleep apnea: a double-blind, placebo-controlled study. Pediatrics.

